# Assessing coastal vulnerability to erosion and storm surges: The case of Richards Bay, South Africa

**DOI:** 10.4102/jamba.v18i1.2119

**Published:** 2026-07-30

**Authors:** Suvana Alakram, David W. Hedding

**Affiliations:** 1 Department of Geography, College of Agriculture and Environmental Sciences, University of South Africa, Johannesburg, South Africa

**Keywords:** coastal erosion, shoreline dynamics, storm surge, coastal zone management, Digital Shoreline Analysis System, South Africa

## Abstract

Sandy beaches are dynamic in nature and subject to natural and anthropogenic processes that influence their evolution. One of the key drivers of coastal evolution is coastal erosion. Sandy beaches are more susceptible to erosion because their fine sand is easily erodible. As approximately 80% of South Africa’s coastline consists of sandy beaches, including environmentally sensitive dune systems, an impetus is to improve our knowledge and understanding of processes in the coastal zone to aid in coastal zone management and planning for future hazards. This research integrated geographical information systems, remote sensing and modelling techniques to determine the interrelationships between the spatial and temporal dynamics of shoreline change along the Richards Bay coastline in South Africa. Shoreline positions were quantified over 45 years between 1977 and 2022 using the United States Geological Survey’s Digital Shoreline Analysis System. The historical data were then used to estimate shoreline positions 10 years and 20 years into the future using the extrapolated linear regression method. The study revealed persistent erosion north of the harbour, with accretion occurring south of the harbour. Furthermore, the storm surge analysis revealed that a 10 m surge inundated 82% of the study area.

**Contribution:**

This serves as an important source of information for flood risk analysis and disaster management within and around the Port of Richards Bay, South Africa.

## Introduction

Human population density is increasing worldwide, especially in coastal areas, which offer abundant scenic beauty, an idyllic climate, economic growth and recreational activities. Consequently, the vulnerability of coastal areas to anthropogenic changes is increasing. Currently, it is estimated that approximately 40% of the world’s population resides within 100 km of the coast (United Nations 2017). As such, the exploitation of coastal regions for urban development and tourism activities results in a phenomenon known as ‘coastal squeeze’. This process of coastal squeeze reduces the area available for natural processes to occur, thereby making the coastline vulnerable to increased erosion (KwaZulu-Natal Department of Economic Development, Tourism, and Environmental Affairs [KZN EDTEA] 2023), which will be further intensified by rising sea levels linked to climate change. Erosion is the removal and deposition of soil, rock or dissolved particles from one place to another and occurs across all landscapes on Earth’s surface. Coastal erosion is a process where the shoreline retreats in a landward direction because of a lack of sediment on a beach (Corbella & Stretch 2012; Esterhuizen 2019; Murray et al. 2023; Smith et al. 2014). Coastal erosion results from a combination of natural and anthropogenic factors. Natural forces include wave regimes, ocean currents, tides and sediment dynamics; anthropogenic activities such as harbour construction, sand mining and tourism developments can cause significant changes to the coastal landscape. For example, the sand on the beaches of Richards Bay, South Africa, originates from inland erosion by the Thukela (previously Tugela), uMhlathuze and Mlalazi Rivers and is washed onto the beaches by waves (Simmonis 2016). Consequently, sand mining in these rivers is impeding sand delivery to the coast, exacerbating erosion. Regionally, agricultural expansion and dam construction are also affecting sediment transport to the coast, thereby increasing coastal erosion. Other factors that also impact coastal erosion include the nature and source(s) of the sand, that is, how easily it is eroded, and local coastal geomorphology (Simmonis 2016).

Managing coastal environments to mitigate coastal erosion requires knowledge of the various processes that drive coastal evolution. This knowledge is critical for categorising the different processes underlying coastal evolution (Palalane 2016). Monitoring shoreline position has become an effective approach for assessing the dynamics of coastal erosion and coastline evolution. Shoreline studies are conducted using remote sensing data and analysis techniques in a Geographic Information System (GIS) at different spatial and temporal scales. Globally, shoreline dynamics have been addressed by numerous studies (Baig et al. 2020; Bheeroo et al. 2016; Ferreira et al. 2021; Hossen & Sultana 2023; Kilar 2022; Mutaqin 2017; Oyedotun 2014) and in South Africa (Goble & MacKay 2013; Murray et al. 2023; Smith et al. 2014). Remote sensing technologies, including satellite imagery, aerial photography, airborne light detection and ranging (LiDAR) sensors and synthetic aperture radar (SAR) sensors, provide reliable data for monitoring shoreline changes (Ferreira et al. 2021; Kilar 2022). The analysis of satellite imagery is the most commonly used technique for monitoring shoreline changes (Kilar 2022; Tsai 2022). In addition to using shoreline geometry to measure rates of change over time, historical shoreline geometry data can also be used to predict future shoreline positions, which is vital in the context of climate change and sea level rise. Thus, this study aims to investigate historical shoreline changes and predict the future range of recession to support coastal zone management in Richards Bay, South Africa. It also aims to assess the vulnerability of the study area to storm-surge inundation using model-based approaches.

Typically, erosion rates are determined by performing a linear regression analysis and using the slope of the fitted line to extrapolate into the future, thereby forecasting future shoreline positions based on the linear behaviour of past shorelines. This type of time-series forecasting has some limitations, as it does not account for relevant processes occurring along the coast. For example, accelerated erosion from storm surges, the addition of coastal erosion infrastructure, such as groynes, interruptions in sediment transport or sea-level rise (Calkoen et al. 2021). Even so, some states in the United States implicitly use this forecasting method to implement regulations based on 30-year erosion rates (Farris, Long & Himmelstoss 2023). In South Africa, this type of forecasting technique is also used by some municipalities to develop risk setback lines (Goble & McKay 2013). Coastal risk setback lines are defined by a specific distance from the shoreline where development is prohibited to protect the coastline. In South Africa, the *Integrated Coastal Management Act* (ICM), *Act 24 of 2008*, refers to these as ‘Coastal Management Lines’. It is defined as ‘designated boundaries along the coastline that mark the limit of development to protect coastal areas from natural hazards like erosion, flooding, and sea level rise’.

The effects of climate change are felt more rapidly in coastal areas, where sea level rise poses a challenge to coastal management. These climate change impacts are expected to increase the exposure of coastal infrastructure and communities to storm-surge hazards, underscoring the urgent need to study and understand the effects of storm surges on coastal zones (Kim et al. 2021). For municipality planners and disaster management agencies to effectively plan and prepare for flooding caused by storm surges, information on inundated areas is required (Balstrom & Kirby 2022). The modelling of extreme weather events, such as storm surges, is primarily centred on hurricane-induced models based on empirical studies, considering parameters such as wind, atmospheric pressure, waves, the geomorphology of the coastline, geology and bathymetry (Balstrom & Kirby 2022). In lieu of this, Balstrom and Kirby (2022) presented a similar but simpler application for modelling storm surges, in which predictions are based on digital elevation data and assume steady-state terrain conditions. This approach is used to investigate historical shoreline changes and predict the future range of recession, aiding coastal zone management. It also aims to assess the vulnerability of the Port of Richards Bay, South Africa, to storm-surge inundation using model-based approaches.

## Research methods and design

**FIGURE 1:**
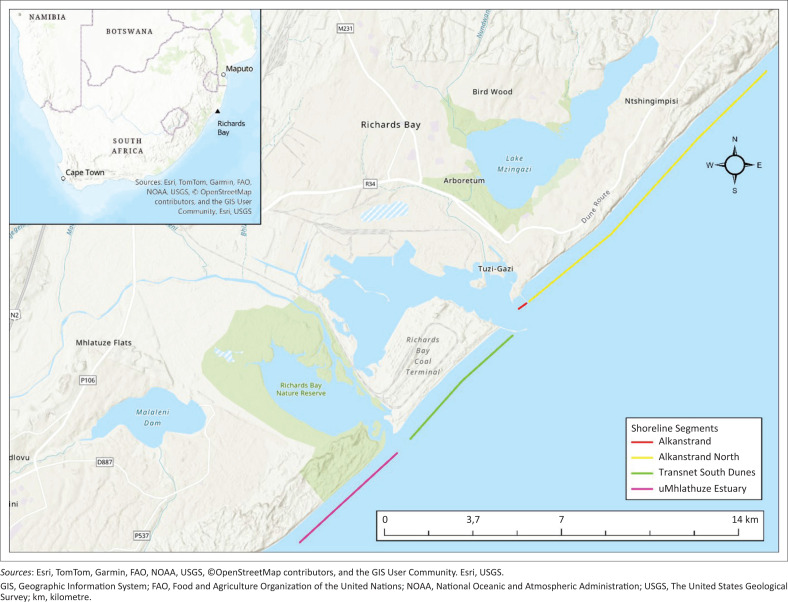
Locality map showing the coastline and surrounding areas of the Port of Richards Bay, South Africa.

The coastal town of Richards Bay, South Africa, has 48 km of coastline, 80% of which is in its natural state, and is characterised by sandy beaches backed by well-established dune formations (uMhlathuze Municipality 2022). The study area covers a 21-km stretch of coastline in Richards Bay, which has been divided into four beach areas under investigation as illustrated in Figure 1. These beaches are separated by breakwater dolosse and a river mouth, which could not be analysed as a single stretch of coastline in the Digital Shoreline Analysis System (DSAS). The beaches south of the harbour are the uMhlathuze Estuary, measuring 6.8 km, and the South Dunes, measuring 4.3 km. The beaches north of the harbour are Alkanstrand, a relatively small beach measuring 0.4 km, and Alkanstrand North, measuring 10.4 km. Both Alkanstrand and Alkanstrand North constitute the main recreational beach areas, whereas South Dunes is by permit only, and the uMhlathuze Estuary is not accessible to the public. Observed erosion has occurred mainly at Alkanstrand North, which will be the focal point of the analysis in terms of erosion.

The DSAS was used to estimate shoreline movements in the study area over time. To do this, the DSAS measures changes in shoreline positions over time to compute a range of statistical parameters. Shoreline rates of change for the study area were calculated by digitising shoreline positions across eight different time periods from 1977 to 2022 over 45 years. After preparing the data in ArcGIS Pro, it was converted to a format accepted by DSAS version 6 (i.e. GeoJSON format). The DSAS was then used to calculate various rates of change based on measured differences between shoreline positions through time, incorporating a relative level of uncertainty. Georeferencing and removing distortions from maps and aerial photographs will always introduce associated errors or uncertainties in their positions (Murray et al. 2023). These errors can be attributed to human error, such as inaccurate control points, transformation methods, image resolution or scale (Morton & Miller 2005; Murray et al. 2023). The resultant residual error is the distance between two points (historical image, *x*, and reference image, *y*) and is based on the Pythagorean formula (Equation 1):

$$Square\;Root\;(Residual\;x^{2}+Residual\;y^{2})$$ [Eqn 1]

For this study, the georeferencing errors were noted as being between 5 m and 10 m. Delineation of the shoreline was conducted using the high water line (HWL) as a reference. For this study, the HWL is defined as the point that represents the maximum rise of a body of water above land.

The workflow for shoreline change analysis begins with collecting historical aerial photographs, which are then pre-processed through georeferencing and mosaicing. Shorelines for selected years (i.e. 1977, 1984, 1992, 2004, 2009, 2013, 2016 and 2022) were manually digitised and converted to GeoJSON format using a personal geodatabase. This dataset is analysed with the DSAS, which measures long-term changes from 1977 to 2022 using metrics such as Net Shoreline Movement (NSM), Shoreline Change Envelope (SCE), End Point Rate (EPR), Linear Regression Rate (LRR) and Weighted Linear Regression (WLR). Finally, DSAS extrapolates these trends to forecast shoreline positions for 2032–2042, providing insights about historical changes and future projections for coastal management.

Between 1977 and 2022, eight shoreline datasets were compiled from both National Geospatial Information (NGI) and Google Earth sources, each with varying scales and resolutions. The earliest historical records from 1977, 1984 and 1992 were captured by NGI at scales of 1:30 000, 1:20 000 and 1:50 000, respectively, with between three and seven photographs per dataset. Later records from 2004 to 2022 were sourced from Google Earth at a finer 1:10 000 scale, each supported by five photographs. From 2009 onwards, NGI provided higher-resolution 0.5 m georectified imagery, with five to six photographs per dataset in 2009, 2013 and 2016. This progression reflects a shift from broader-scale aerial photography to more precise, high-resolution georectified imagery, ensuring consistent shoreline monitoring across multiple decades. The time series for this dataset was primarily chosen based on the development of the harbour in 1976, noting the disturbance of the natural system. The aerial photographs were obtained from the Directorate: NGI, a division of the Department of Agriculture, Land Reform and Rural Development (DALRRD). The historical aerial photographs from 1977, 1984 and 1992 were scanned as black-and-white images with their associated metadata. These were rectified and georeferenced to align with the Environmental Systems Research Institute’s (ESRI) ArcGIS Pro imagery basemap (2019) and projected to World Geodetic System (WGS) 1984 Universal Transverse Mercator (UTM) Zone 36S. Ground control points were identified in each of the 40 aerial photographs and linked to the basemap layer, used as the reference image. The photographs were transformed using a first-order polynomial in ArcGIS Pro. The georeferenced images were then combined using the mosaic tool, thereby creating a single raster dataset for each year. A total of eight shoreline positions from different years were analysed for rates of shoreline change.

The DSAS generates transects that are cast perpendicular to the reference baseline at 50-m intervals. Then, it measures the distance between the baseline and each shoreline intersection along a transect and combines timeline information and positional uncertainty of each shoreline to generate change statistics and distance measurements. Shoreline forecasting was conducted using a linear regression of the available number of shoreline positions to calculate a rate of change. This rate of change was then used to ‘extrapolate a linear regression into the future’ (Farris et al. 2023:1). The extrapolated linear regression (ELR) method is a statistical technique for predicting values outside the observed data range. It assumes that the relationship between two variables remains constant and linear even beyond the observed range. Thus, the predicted shoreline position (*Y*_f_) given by Farris et al. (2023) (Equation 2) is:

$$Y_{f}=m\cdot X_{s}+b$$ [Eqn 2]

where:

*m* – the rate of shoreline change

*X_s_* – date of the latest shoreline

*B* – *y*-intercept

For the *m* value, the LRR is taken from the results of the DSAS analysis for the shoreline change analysis, along with its associated uncertainties. The year is expressed as a decimal year. The formulas were computed in Microsoft Excel to extrapolate future shoreline positions 10 years and 20 years into the future.

To assess storm-surge inundation in the study area, a 5 m digital elevation model (DEM) was used from the Centre for Geographical Analysis (CGA) at Stellenbosch University. This high-resolution DEM was hydro-conditioned to provide flow paths representing water flowing beneath bridges, roads and so on. The digitised hydro-adaptations were ‘burned’ onto the DEM using the ‘Burn Flowlines onto Digital Terrain Model (DTM)’ tool created by Balstrom and Kirby (2022). The DEM with hydro-adaptations was then used in Model Builder in ArcGIS Pro to identify floodable areas. Using the 5 m DEM, a source line feature representing the boundary of the study area and desired storm-surge levels, the model assesses how water inundates the study area. Surge levels (7 m and 10 m) were used for the assessment. A 5 m DEM was used to create the DhyMSea layer, which is a hydro-conditioned version of the DEM. The 5 m DEM means that each cell represents a 5 m by 5 m square on the ground. Importantly, each raster cell represents the elevation above mean sea level in metres.

### Ethical considerations

Ethical clearance for the study was acquired from the Ethics Committee of the University of South Africa (Reference: Ethics_2023_CAES_HREC_2630).

## Results

The uMhlathuze Estuary, Transnet South Dunes, Alkanstrand and Alkanstrand North shorelines all experience micro-tidal conditions but differ in length, morphology and infrastructure. The uMhlathuze Estuary spans 6.8 km with sandy beaches backed by high vegetated dunes in a largely natural state, while the 4.3 km South Dunes section also features vegetated dunes but includes breakwater dolosse to the north. Alkanstrand is much shorter at 0.4 km, characterised by a steep sandy beach with small, eroded dunes and breakwater dolosse on either side. In contrast, Alkanstrand North extends 10.4 km, marked by sea cliffs topped with dunes over semi-consolidated sediment, with dolosse to the south and irregular sand nourishment further north. Each site was analysed using eight shoreline positions across identical time intervals from 1977 to 2022. The results of the shoreline change analysis, shoreline forecasting and the inundation modelling (storm surge) are presented here.

### Shoreline change

**FIGURE 2:**
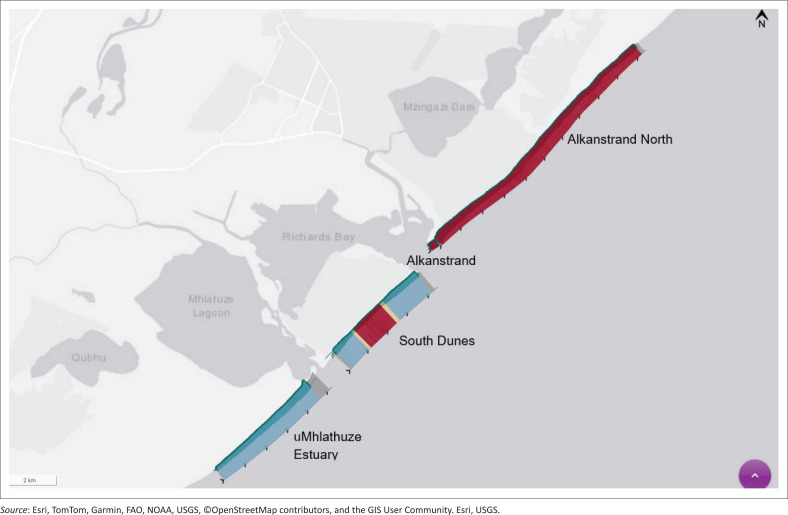
Summary of the results map from Digital Shoreline Analysis System showing erosion in red, accretion in blue and no change in yellow.

The high-level map of the DSAS results indicates that erosion is occurring north of the harbour while accretion is predominantly occurring south of the harbour (Figure 2). Table 1 shows the rate of change statistics calculated by the DSAS, i.e. linear regression, WLR and EPR. Table 2 and Table 3 summarise the distance measurements, i.e. NSM and SCE.

**TABLE 1:** Summary of the erosion and accretion at the four beaches using linear regression, weighted linear regression and end point rate.

Shoreline	Method	Average erosion rate (m/yr)	Percentage of erosional transects (%)	Maximum value erosion (m/yr)	Average accretion rate (m/yr)	Percentage of accretional transects (%)	Maximum value accretion (m/yr)
uMhlathuze Estuary	Linear regression	−0.13	3.3	−0.17	2.33	96.8	5.46
	Weighted regression	−0.33	16.3	−0.67	2.44	83.7	4.86
	End point rate	−0.04	1.6	−0.06	2.10	98.4	4.09
South Dunes	Linear regression	−3.25	61.5	−4.84	1.32	38.5	2.17
	Weighted regression	−2.35	56.4	−3.50	1.74	43.6	2.92
	End point rate	−5.18	59.0	−7.75	2.09	41.0	3.51
Alkanstrand	Linear regression	−3.25	100.0	−3.48	0.00	0.0	0.00
	Weighted regression	−2.93	100.0	−3.03	0.00	0.0	0.00
	End point rate	−3.73	100.0	−3.91	0.00	0.0	0.00
Alkanstrand North	Linear regression	−2.28	100.0	−4.73	0.00	0.0	0.00
	Weighted regression	−2.22	100.0	−5.37	0.00	0.0	0.00
	End point rate	−2.18	100.0	−4.86	0.00	0.0	0.00

m, metres; yr, year.

**TABLE 2:** Summary of the net shoreline movement, with landward movement as negative and seaward movement as positive.

Shoreline	Average distance (m)	Net erosion (%)	Maximum erosion (m)	Net accretion (%)	Maximum accretion (m)
uMhlathuze Estuary	93.06	1.63	−2.65	98.37	183.97
South Dunes	98.90	58.97	−348.84	41.03	157.87
Alkanstrand	−167.80	100.00	−176.09	0.00	0.00
Alkanstrand North	−98.20	100.00	−218.80	0.00	0.00

m, metres.

**TABLE 3:** Summary of the shoreline change envelope, positive in either direction.

Shoreline	Average distance (m)	Maximum distance (m)	Minimum distance (m)
uMhlathuze Estuary	136.98	302.33	57.50
South Dunes	193.10	363.06	45.48
Alkanstrand	169.74	184.52	149.73
Alkanstrand North	123.88	218.80	70.77

m, metres.

The LRR uses all shoreline dates to calculate linear trends over time using the least sum of squares method. The LRR is therefore the slope of the line. The LRR for Alkanstrand and Alkanstrand North revealed 100% erosional transects, with an average erosion rate of −3.25 m and −2.28 m per year, respectively, over the 45-year analysis period. In contrast, the uMhlathuze Estuary had 96.75% accretional transects with an average erosion rate of 0.13 m per year, while the South Dunes showed an average erosion rate of −3.25 m per year. This is illustrated in Figure 4.

The WLR assigns different weights to data points based on their uncertainty values. Uncertainty in shoreline positions arises from various factors, including digitising errors, data quality, georeferencing errors and other uncertainties. Although the WLR is generally considered more reliable than the LRR because it accounts for data uncertainty, the LRR method was selected to assess the shoreline change rate (in this study) because it is used in DSAS version 6 (The United States Geological Survey [USGS], Reston, VA, US). Farris et al. (2023) highlight that linear regression provides a valid formula for shoreline forecasting.

The EPR is another statistical method that the DSAS uses to calculate shoreline change. It is calculated by dividing the total distance of shoreline movement by the time elapsed between the oldest and most recent shorelines, yielding a rate in metres per year. Positive values indicate accretion, while negative values indicate erosion. The results of the EPR were similar to the LRR, with average erosion rates of −3.73 m and −2.18 m per year at Alkanstrand and Alkanstrand North, respectively. Similarly, uMhlathuze and South Dunes reported an average accretion rate of 2.44 m and 1.74 m per year, respectively.

The NSM represents the distance between the oldest and the most recent shorelines. uMhlathuze Estuary and South Dunes, situated South of the Port of Richards Bay, experienced 98.37% and 41.03% accretion, with an average seaward expansion of 93.06 m and 98.90 m, respectively. While Alkanstrand and Alkanstrand North, situated North of the Port of Richards Bay, experienced 100% erosion, with an average landward retraction of −167.80 m and −98.20 m, respectively.

The SCE value represents the greatest distance among all the shorelines that intersect a given transect, regardless of date or direction (i.e. the distance between the most landward and most seaward shorelines). For the uMhlathuze shoreline, all 123 transects differed from that of the NSM value. This means that the shoreline position has shifted back and forth or undergone significant changes in different directions over time, resulting in a wider range of movement captured by the SCE than by the NSM.

### Shoreline forecasting

**FIGURE 3:**
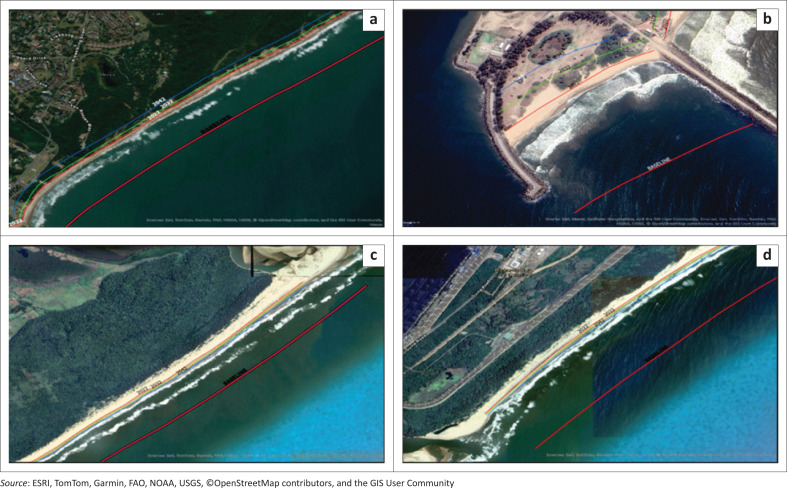
Predicted shoreline positions for 2032 (green) and 2042 (blue) with 2022 (red) as a reference point. (a) Alkanstrand North, (b) Alkanstrand, (c) uMhlathuze Estuary and (d) South Dunes.

Shoreline positions were predicted for 10 years and 20 years (i.e. 2032 and 2042) into the future, based on the ELR method and formula given by Farris et al. (2023). The predicted shorelines for 2032 and 2042 are visualised in Figure 3.

The results show that for Alkanstrand North, the shoreline will erode by a further 10% by 2032 and 23% by 2042 compared with the most recent shoreline (2022). These results assume that the linear trend continues. In line with the erosion trend, the 400-m stretch of Alkanstrand beach predicted a 21% increase by 2032 and a 36% increase by 2042 compared with the most recent shoreline (2022). The analysis does not consider the future effects of sea level rise, climate change-induced weather events such as storm surges and high-intensity storms or other geomorphological events. Therefore, it is assumed that these figures are underestimates and might be higher than predicted.

Conversely, the results for South Dunes revealed a slight 2% increase in accretion by 2032 and 4% by 2042, assuming the linear accretion trend continues. Forecasting for the uMhlathuze Estuary by 2032 shows a predicted seaward movement of the shoreline of 0.6% and 1.7% by 2042. It is noticeable that the rate of erosion is much higher to the North of the Port of Richards Bay than the rate of accretion to the south.

### Assessment of storm surges

**FIGURE 4:**
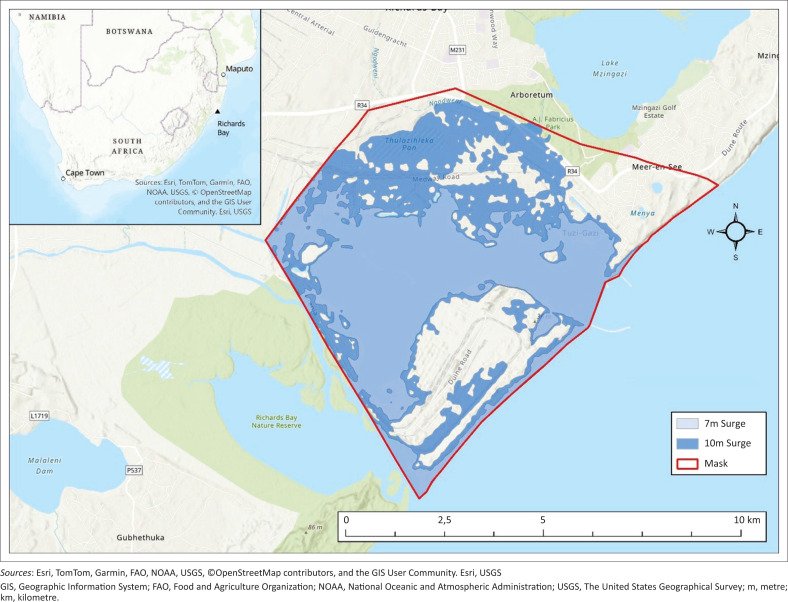
Inundation model depicting the floodable areas of the study area when experiencing a storm surge level of 7 m and 10 m.

Two levels of storm surge were analysed to assess their potential impact on the coastal region of Richards Bay (i.e. 7 m and 10 m). The chosen surge levels of 7 m and 10 m were used in the analysis to replicate previously recorded data. Smith et al. (2010) indicated that large swells of 8 m – 10 m are commonly recorded during mid-winter storms in the KwaZulu-Natal Bight. The maps in Figure 4 were generated from the results of the storm surge inundation exercise at levels of 7 m and 10 m.

The results of the storm surge analysis reveal that, for a 7 m storm surge, the study area is minimally affected with a 5.2% expansion of water extent. However, at a surge level of 10 m, a significant portion of the modelled study area is inundated (i.e. 82%). At the 10 m modelled surge level, most buildings and infrastructure are affected, except in high-elevation areas. This is an important source of information for flood risk analysis within the Port of Richards Bay and can serve as a guide for designing mitigation measures to limit inundation of affected buildings and infrastructure. Such measures can include strengthening or refurbishing existing breakwaters to better cope with changing conditions under climate change. Information about the inundations because of storm surges can also be useful in rescue and damage assessments (Balstrom & Kirby 2022).

Evidence has shown that the coastline around Richards Bay will become more vulnerable to tropical storms and cyclones (Nathaniel & Baxter 2025). Temperatures in South Africa are expected to increase by up to 4°C by 2100 (Fitchett, Grant & Hoogendoorn 2016). In addition to changes in wind direction and speed, the severity of precipitation events and rising sea levels are expected (Fitchett et al. 2016). Sea level rise also increases the risk of storm surges in low-lying areas. Thus, the likelihood that the study area will experience a 10 m storm surge level or higher increases.

## Discussion

The results of this study demonstrate a distinct spatial variation in shoreline behaviour along the Richards Bay coastline, with significant erosion on beaches north of the harbour and measurable accretion on beaches south of the harbour. This pattern is consistent with the predominant south-to-north longshore sediment transport regime along the KwaZulu-Natal coastline (Cooper 1991; Simmonis 2016; Smith, Bundy & Cooper 2016). The Richards Bay harbour breakwaters have interrupted the natural littoral drift, resulting in sediment accumulation on the updrift southern side and sediment depletion, and consequent erosion, along the downdrift northern beaches. Similar findings were reported by Smith et al. (2016), who identified the Richards Bay and Durban breakwaters as significant contributors to downdrift coastal erosion, despite ongoing inadequate sand nourishment interventions. Furthermore, Wells (2015) modelled alternative sand nourishment schemes for Richards Bay to mitigate erosion resulting from the disruption of longshore sediment supply caused by harbour infrastructure. Collectively, these findings support established coastal geomorphological theory and reinforce the conclusion that the harbour breakwaters are the primary anthropogenic driver of present-day shoreline morphology and sediment dynamics along this section of the coastline.

Other contributing factors leading to coastal erosion along the coastline of Richards Bay are linked to activities in the hinterland, such as sand mining in rivers, river impoundment and climate-related impacts such as sea-level rise and the increase and intensity of storm surges (KZN EDTEA 2023; Luck-Vogel, Le Roux & Ludick 2019). Should erosion rates and extreme weather events continue, the shoreline will retreat, leading to further loss of natural habitats, coastal dunes, associated vegetation and infrastructure.

### Proposed strategies to mitigate coastal erosion

Wells (2015:2) emphasises the importance of a well-executed sand bypassing scheme for the study area and, as such, has stated that ‘implemented correctly, sand nourishment schemes can be a cost-effective solution to coastal vulnerability with environmental and economic benefits while reducing storm damage’. Another benefit of beach nourishment via a sand-bypassing scheme is that the sediment collected and deposited is of the same grade, and no foreign material is introduced into the marine system, which provides an added environmental incentive.

In 2019, Transnet National Ports Authority (TNPA) announced the implementation of its unique sand-bypassing scheme at the Port of Ngqura (Gqeberha), making it the first port in the world to have a fixed jet-pump sand-bypassing system that mimics natural longshore drift (TNPA 2019). Should this system prove economically viable at the Port of Richards Bay, it would stabilise the shoreline, promote the recovery of environmentally sensitive dunes and their vegetation and help the beaches cope with storm-surge impacts.

The role of risk setback lines is outlined in the *ICM (Act No 28 of 2008)* as amended. The *ICM Act* identifies a setback line as a line ‘seawards of which development can be prohibited or controlled’ (Goble & MacKay 2013:2125). In South Africa, there is no legislative requirement for setback lines, delineation or undertaking. As such, this study uses the 2032 and 2042 predicted shoreline positions, as calculated by DSAS and the ELR method, to demarcate risk setback lines for the study area. The indicated shoreline positions at 2032 and 2042 can be regarded as buffer zones where no development should occur. The establishment of these setback lines can prove useful in coastal development planning and disaster management planning. Farris et al. (2023) have noted that some states in the United States primarily use setback lines associated with 30-year erosion rates extrapolated into the future as a basis for regulations.

Geotextile sandbags can be effective when implemented correctly although they are not a long-term erosion protection measure. Geotextile bags have been implemented as a coastal erosion strategy in the study area since 2015. Intense storm surges, bringing high-energy waves and wind, shift the bags and erode the sand beneath them. Rock revetments were installed in the study area in 2024, and their success is yet to be determined. The addition of breakwater dolosse along the northern beaches can stabilise the beach by acting as a system that produces a domino effect. Lastly, a retreat can be initiated to relocate existing structures to less vulnerable areas.

## Conclusion

It can be stated that the coast will erode, and the shoreline will eventually recede. We need to know the volume of erosion and the extent of expected recession to prepare a sustainable solution for coastal risk reduction. The study found that large-scale erosion is taking place north of the harbour because of disruption of the longshore sediment pattern (Schoonees & Theron 2002; Simmonis 2016; Wells 2015), lack of sediment supply (Breetzke, Moore & Celliers 2016; Esterhuizen 2019) and severe weather events (Smith et al. 2010). Coastal dunes act as a natural barrier against storm surges and coastal inundation. It plays a key role in stabilising the coast and serves as an essential sediment reserve for beaches (Flor-Blanco et al. 2021). Rising sea levels driven by climate change, however, introduce uncertainty about increased storminess, which needs active monitoring of beach and dune erosion.

The purpose of this research was to monitor historical shoreline changes along the coastline of Richards Bay and to predict its future position using remote sensing and GIS technologies. The DSAS software was utilised to calculate shoreline change rate statistics over a 45-year period (1977–2022). This data were used to predict its position 10 years and 20 years into the future using the ELR statistical method. In response to the increasing frequency and intensity of storm surges in recent years, a storm-surge inundation screening exercise was conducted to model water flow paths at surge levels of 7 m and 10 m. The results are expected to aid in disaster risk planning and management in the coastal zone. Recommendations were proposed to help mitigate coastal erosion in the study area. These site-specific strategies are based on the study results and the landscape’s geomorphological features. Although erosion cannot be completely stopped, it can be significantly reduced through the implementation of the proposed mitigation measures.
